# Supplementary material for: Chromosome architecture in an archaeal species naturally lacking structural maintenance of chromosomes proteins
Source: Nat Microbiol. 2023 Dec 18;9(1):263–73. doi: 10.1038/s41564-023-01540-6 (PMC10769869; doi:10.1038/s41564-023-01540-6)
Supplement: Supplementary file 1 — Supplementary Figs. 1–4 and Table 1. [file 41564_2023_1540_MOESM1_ESM.pdf]

nature microbiology

Article

https://doi.org/10.1038/s41564-023-01540-6

Chromosome architecture in an archaeal species naturally lacking structural maintenance of chromosomes proteins

In the format provided by the authors and unedited

Supplementary Information

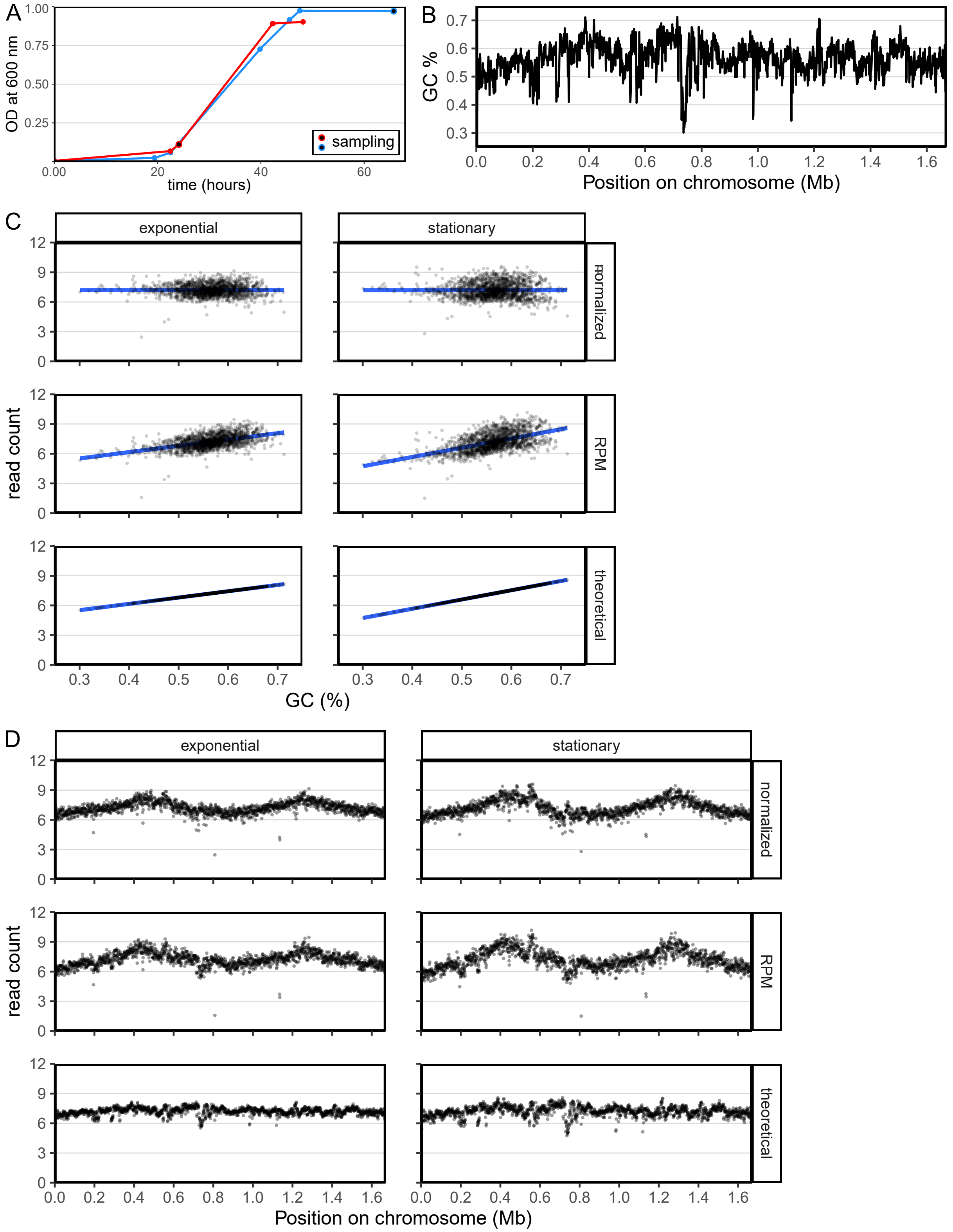

Supplementary Figure 1. Normalization of the Marker Frequency Analysis. A. OD600 at the sampling points. The red and blue curves corresponds to the exponential and stationary phase samples, respectively. B. GC content in 1 kb non overlapping windows. genome. C. GC-normalized, RPM-normalized

(read per million reads) and HC-modelled (theoretical) read count in function of the GC content for exponential and stationary phase. D. GC-normalized, RPM-normalized (read per million reads) and HC-modelled (theoretical) read count along the chromosome for exponential and stationary phase.

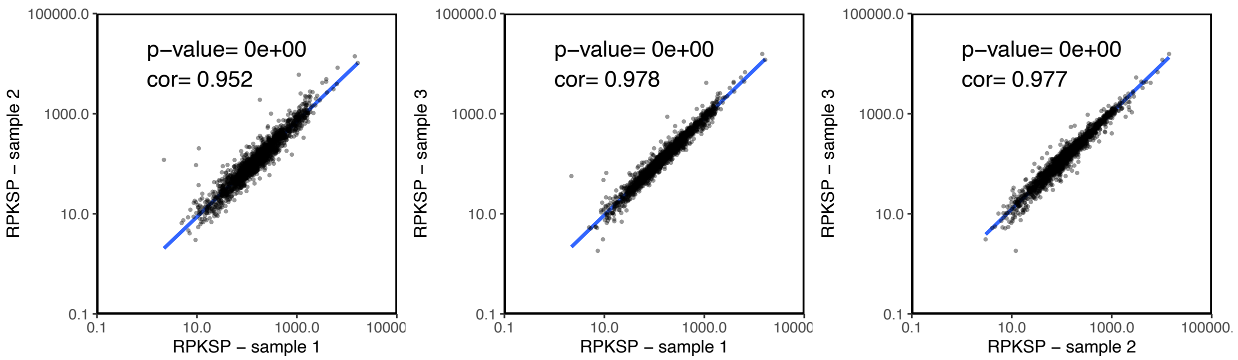

Supplementary Figure 2. RNAseq reproducibility in stationary phase between the three samples. Two-sided Pearson correlation p-value and coefficient are indicated.

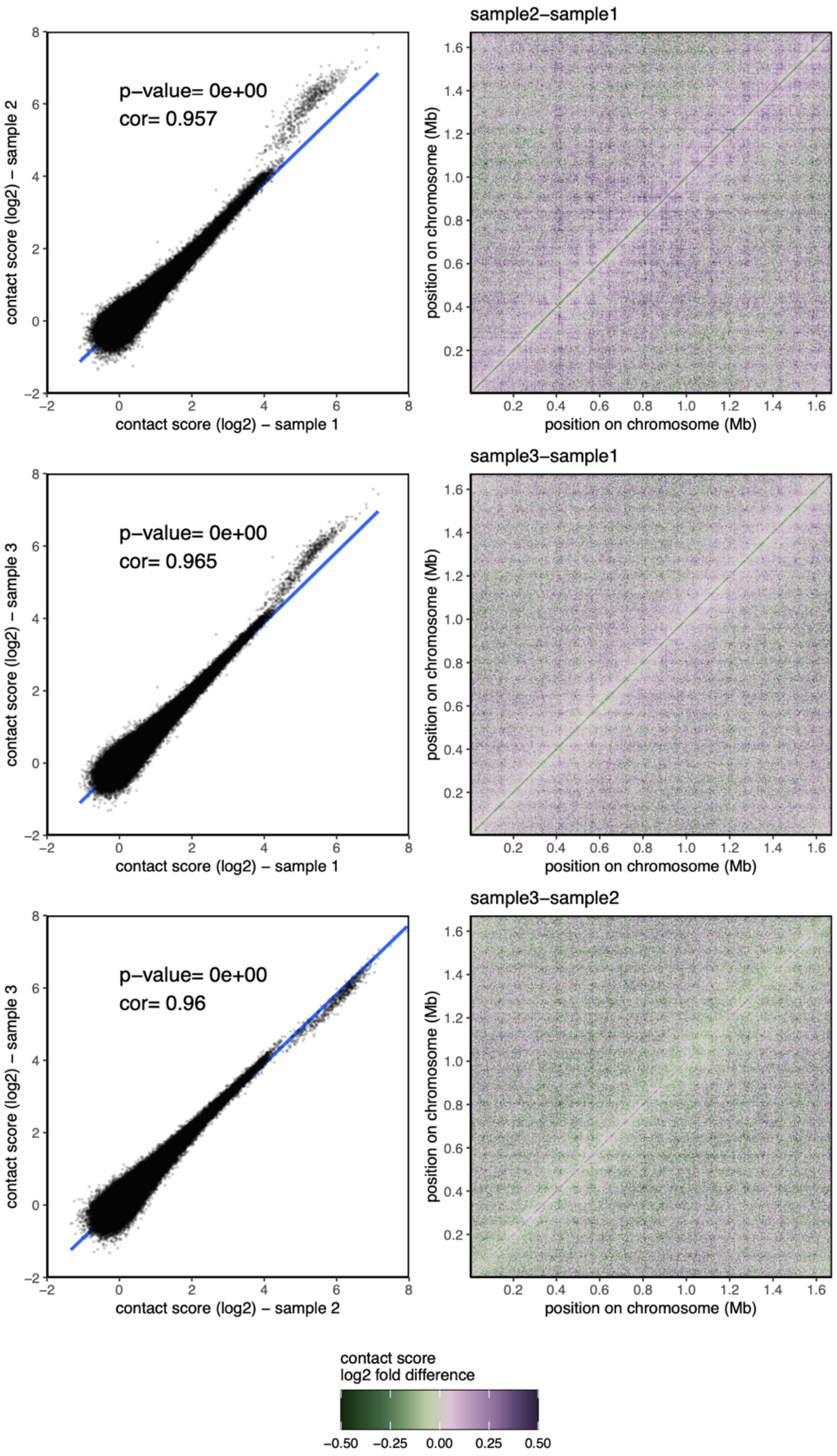

Supplementary Figure 3. 3C-seq reproducibility in stationary phase between the three samples: pairwise contact score comparison and Log2 Fold Difference heatmap. The Pearson correlation p-value and coefficient are indicated.

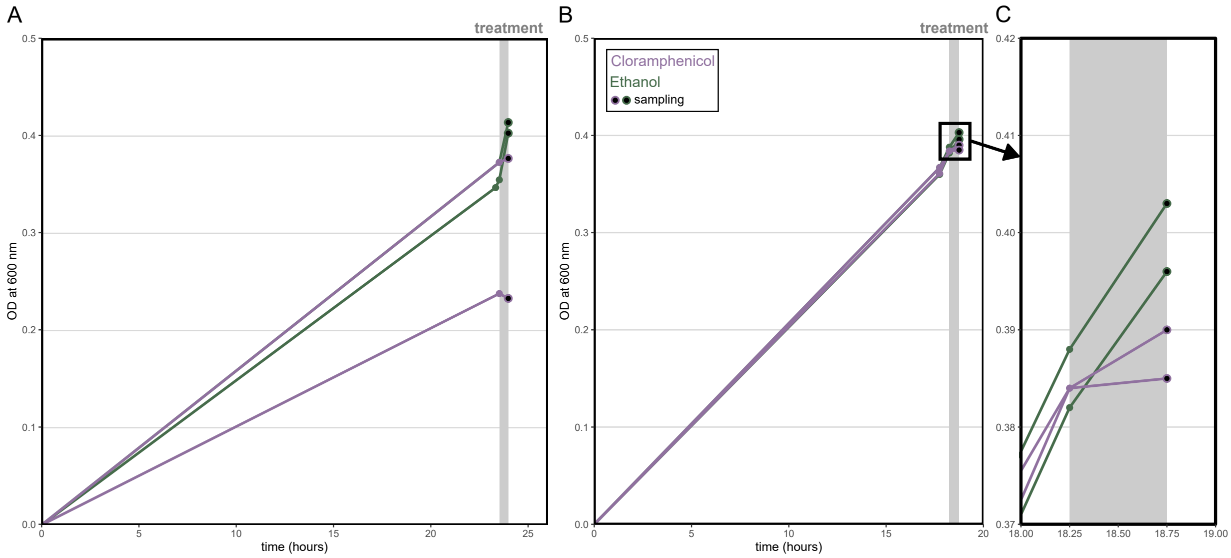

Supplementary Figure 4. Sampling time points for the transcription reconfiguration (A) and translation reconfiguration (B) experiments. C. For the translation reconfiguration experiment, a more detailed scale is shown. The 30min treatment period is indicated with a grey background.

Supplementary Table 1. Genomes used in this study.

Species	Assembly Accession	Core dataset
Aeropyrum pernix K1	NC_000854.2	A[1], AA[2], AAP[3], AAPD[4]
Aeropyrum camini SY1	NC_022521.1	A, AA, AAP, AAPD
Acidolobus saccharovorans 345-15	NC_014374.1	AA, AAP, AAPD
Acidolobus sp. 7A	NZ_CP010515.1	AA, AAP, AAPD
Caldisphaera lagunensis DSM 15908	NC_019791.1	AA, AAP, AAPD
Hyperthermus butylicus DSM 5456	NC_008818.1	AAP, AAPD
Pyrodictium delaneyi Hulk	NCQP01000001.1	AAP, AAPD
Pyrodictium delaneyi Su06	NZ_CP013011.1	AAP, AAPD
Pyrodictium occultum PL-19	LNTB01000001.1	AAP, AAPD
Pyrolobus fumarii 1A	NC_015931.1	AAP, AAPD
Desulfurococcus amylolyticus strain 1221n	NC_011766.1	AAPD
Desulfurococcus amylolyticus strain DSM 1653	NC_018001.1	AAPD
Desulfurococcus mucosus DSM 1653	NC_014961.1	AAPD
Ignicoccus hospitalis KIN4I	NC_009776.1	AAPD
Ignicoccus islandicus DSM 13165	NZ_CP006867.1	AAPD
Ignisphaera aggregans DSM 17230	CP002098.1	AAPD
Staphylothermus hellenicus DSM 12710	NC_014205.1	AAPD
Staphylothermus marinus F1	NC_009033.1	AAPD
Thermogladius calderae strain 1633	NC_017954.1	AAPD
Thermosphaera aggregans DSM 11486	NC_014160.1	AAPD
Thermosphaera aggregans strain 3507	NZ_CP063144.1	AAPD

[1] *Aeropyrum* only
[2] *Aeropyrum* and *Acidolobus*
[3] *Aeropyrum*, *Acidolobus* and *Pyrodictiaceae*
[4] *Aeropyrum*, *Acidolobus*, *Pyrodictiaceae* and the rest of the *Desulfurococcaceae*
